# What factors influence sexual and reproductive health care among adolescent and young adult cancer patients?: a novel serial focus group study

**DOI:** 10.1186/s12885-025-14380-w

**Published:** 2025-07-01

**Authors:** Niki Oveisi, Vicki Cheng, Dani Taylor, Preet Kang, Lori A. Brotto, Stuart Peacock, Helen McTaggart-Cowan, Gillian E. Hanley, Sharlene Gill, Meera Rayar, Amirrtha Srikanthan, Mikaela Barnes, Haydn Bechthold, Norman Jansen, Mary A. De Vera

**Affiliations:** 1 Faculty of Pharmaceutical Sciences, University of British Columbia, 2405 Wesbrook Mall, Vancouver, BC V6T 1Z3 Canada; 2 Collaboration for Outcomes Research and Evaluation, Vancouver, BC Canada; 3 Patient Research Partner, Vancouver, BC Canada; 4 Faculty of Medicine, University of British Columbia, Vancouver, BC Canada; 5 BC Cancer, Vancouver, BC Canada; 6 Faculty of Health Sciences, Simon Fraser University, Burnaby, BC Canada; 7 Faculty of Medicine, University of Ottawa, Ottawa, ON Canada; 8 Department of Medicine, Division of Medical Oncology, The Ottawa Hospital, Ottawa, ON Canada; 9 The Ottawa Hospital Research Institute, Ottawa, ON Canada; 10 Centre for Health Evaluation and Outcome Sciences, Vancouver, BC Canada

**Keywords:** Sexual health, Reproductive health, Cancer survivorship, Adolescent and young adult, Cancer treatment

## Abstract

**Background:**

Disparities in sexual and reproductive health care at diagnosis and during treatment for adolescent and young adult (AYA, ages 15–39) cancer patients may be linked to various factors, including those at the patient- and system-level. We conducted serial focus groups to explore how AYA cancer patients’ experiences with sexual and reproductive health and its care are influenced by their identities (patient factors) and contextual enablers (healthcare factors).

**Methods:**

We recruited individuals aged ≥ 18 years, diagnosed with cancer as an AYA, and residing in Canada. Participants were grouped into cohorts based on identity factors (e.g., gender, cancer stage) with each cohort taking in three serial focus groups mimicking support groups (e.g., sharing information, fostering community). Patient research partners contributed to topic guide development and co-facilitated focus groups. We used framework analysis guided by the PROGRESS-Plus (Place of residence, Race/ethnicity/culture/language, Occupation, Gender, Religion, Education, Social capital, Socioeconomic status, and Plus) framework on health inequity factors and Andersen’s model of access to medical care.

**Results:**

Altogether, 48 participants (age 33 (21–48)) were assigned into eight cohorts. Each cohort took part in three serial focus groups, resulting in a total of 24 focus groups. Among participants, there was representation based on gender (e.g., nonbinary and gender fluid, *n* = 4), non-heterosexual sexual orientation (*n* = 14), and race (*n* = 12). Themes indicate AYA cancer patients’ experiences with lack of sexual and reproductive health information and support, and lack of consideration of experiences and beliefs when providing sexual and reproductive health care. Findings reveal eight identity (patient-level) factors (place of residence, self-advocacy (occupation, education, and plus), socioeconomic status, social capital (plus), gender and sex, age (plus), relationship status (plus), sexual orientation (plus)) and two contextual enablers (health care system-level factors) (inefficiencies and interactions) that influence access to appropriate and available sexual and reproductive health care.

**Conclusion:**

AYA cancer patients experiences with sexual and reproductive health care are influenced by their identity factors and contextual enablers. Treatment plans, research studies, and care programs should account for these to ensure that care is both inclusive and responsive to their unique needs.

**Supplementary Information:**

The online version contains supplementary material available at 10.1186/s12885-025-14380-w.

## Introduction

Cancer incidence has risen by nearly 30% over the last 50 years among adolescents and young adults (AYA), those between 15 and 39 years [1]. While the survival rate for AYA cancer have improved, largely due to advancements in treatments [2], the long-lasting effects of the cancer and its treatment can have persistent effects throughout life [3–5], often leading to ongoing health challenges [6, 7]. Sexual and reproductive health represent some of these enduring challenges [8]. The World Health Organization has defined sexual and reproductive health as “*a state of physical*,* emotional*,* mental and social well-being in all matters relating to: sexuality [sexual health] and the reproductive system and its functions and processes [reproductive health]*” [9]. Addressing these issues is important for AYA cancer patients given that adolescence and young adulthood coincides with the reproductive age range and that cancer and its treatments may affect reproductive and sexual health directly (e.g., through impaired hormonal function) and indirectly (e.g., through body image) [10].

Qualitative research provides contextual understanding AYA cancer patients’ *experiences* with receiving care for reproductive and sexual health. However, the number of such studies is limited, and most have focused on interviews with female AYA cancer patients, highlighting gaps in sexual and reproductive health care for this population. For example, in 2019 Frederick et al. conducted interviews with 23 AYA cancer patients aged 15–25, to explore their perceptions and experiences with sexual and reproductive health communication in oncology settings [11]. Findings highlight the need for improved sexual and reproductive health communication from oncology clinicians, especially when addressing specific sexual and reproductive health issues, and understanding the long-term sexual and reproductive health impacts of cancer [11]. In 2021, Bentsen et al. interviewed 12 female AYA cancer patients regarding reproductive health care during cancer treatment and reported themes related to the need for support for navigating fertility information and inadequate and worrying information causing mistrust [12]. In 2023, Oveisi et al. interviewed 15 female pelvic cancer patients and 9 health care providers and found that most patients had unmet sexual health care needs, largely due to lack of appropriate training for health care providers and limited resources [13]. 

Altogether, prior studies highlight gaps in sexual and reproductive health care experienced by AYA cancer patients. However, it remains unclear from these prior studies how these gaps in sexual and reproductive health care may be influenced by patients’ identities, including factors such as gender, race, and socioeconomic status, which may shape their experiences and access to care. Considering these factors is particularly important given the persistent disparities in cancer survival and outcomes among AYA cancer subgroups. These disparities are especially evident among racial and ethnic minorities [14], individuals with low socioeconomic status (SES) [14, 15], those with mental health diagnoses [15], substance use disorders [15], and sexual and gender minorities [16]. Given the impact of social determinants of health (SDH) on outcomes among AYA cancer patients, it is important to also consider how they influence patients’ experiences with and access to sexual and reproductive health care [17]. Thus, using an intersectional lens, our objective was to employ novel, serial focus groups and explore how AYA cancer patients’ experiences with sexual and reproductive health, and related care, are influenced by their identities (e.g., patient factors) and contextual enablers (e.g., healthcare factors).

## Methods

### Study design

We conducted a qualitative research study within a constructivist paradigm, recognizing that knowledge is shaped by social interactions and is context-specific [18]. In line with calls for patient engagement in research, our research team included patient research partners – individuals with lived experience of AYA cancer who contributed to the study in a role that is distinct from that of research participants [19, 20]. Patient research partners contributed to the study design, co-created the topic guide (Additional file 1), co-facilitated focus groups, and supported with data analysis and interpretation.

### Participants

We recruited participants who were: **(1)** diagnosed with cancer between the ages of 15 and 39; **(2)** at least 18 years old at the time of consent; **(3)** residing in Canada; and **(4)** able to communicate in English. Online social media campaigns using platforms such as Twitter, Instagram, and Facebook were shared by research team members, affiliated organizations, patient research partners, and other organizational partners to maximize reach. Mailing lists were also utilized to reach out to patients from cancer registries. Those interested in participating were directed to the study website, where they could complete an online consent form and questionnaire hosted on the Qualtrics platform [21]. The questionnaire gathered detailed sociodemographic data (e.g., gender, sexual orientation, age, race/ethnicity) and clinical information (e.g., cancer type, stage, age at diagnosis, and treatment history). We employed an iterative purposive sampling approach to ensure a diverse representation of participants.

### Data gathering

We created focus group cohorts based on participants’ shared characteristics collected from the questionnaire, such as age, gender, cancer type, and stage of treatment. Each cohort participated in three sequential focus groups (~ 2 h each) which were designed to mimic cancer support groups in that they brought together individuals with shared experiences (e.g., being an AYA cancer patient) to exchange experiences, information, and support, thus fostering a sense of community [22]. Focus groups were facilitated by a trained facilitator (NO) and a patient research partner with training as a social worker (DT) and explored three main topics: **(1)** reproductive health (e.g., fertility preservation family planning); **(2)** sexual health (e.g., sexual functioning, intimacy); and **(3)** reflections on the care they received for sexual and reproductive health before, during and after cancer treatment. While focus groups are typically held as a single event, our novel approach of conducting serial focus groups offers several advantages. It allows participants to build connections and a sense of community, fosters a supportive environment for discussing sensitive topics, and features shorter individual sessions.

**Fig. 1:**
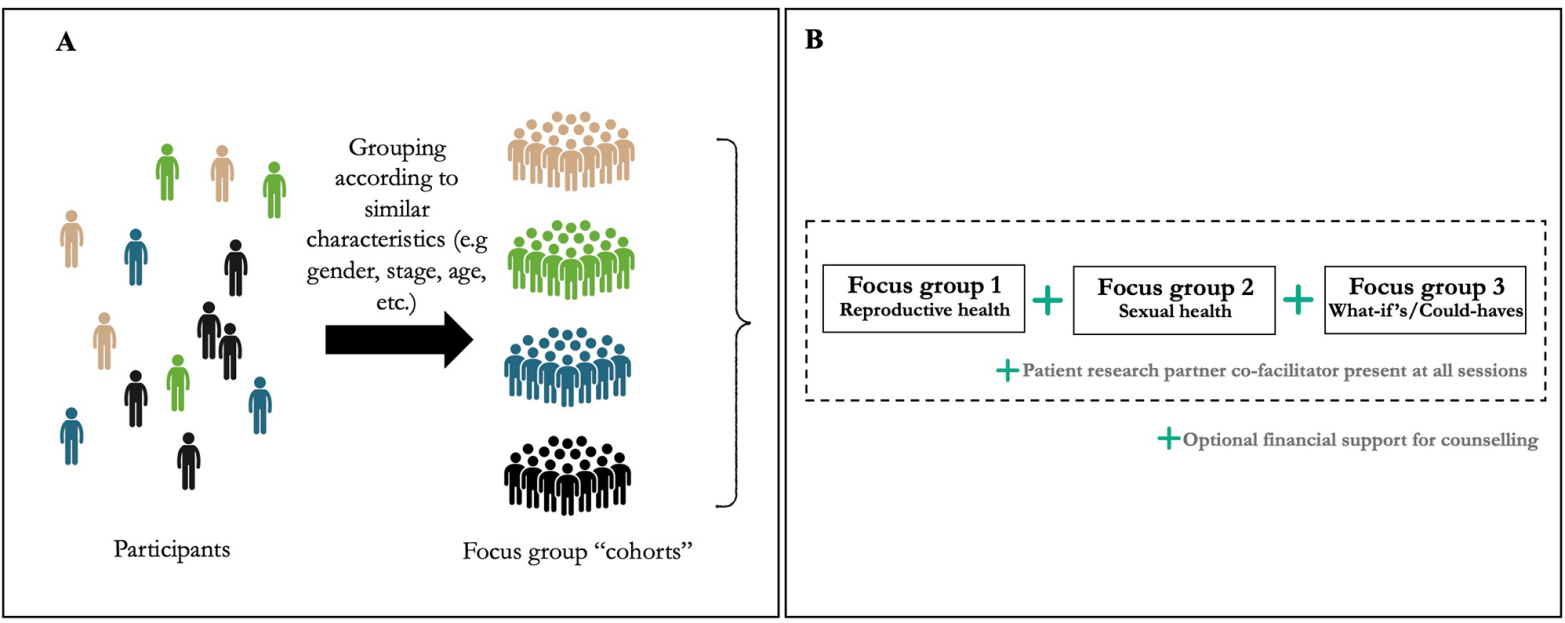
study design including (**A**) Creation of focus groups cohorts and (**B**) Serial focus groups

Focus group cohort creation, data gathering and data analysis occurred simultaneously in an iterative process, with ongoing analysis informing subsequent creation of new cohorts and data gathering until thematic saturation was reached, meaning no new themes or patterns were emerging from the data [23]. Focus groups were conducted on a licensed version of Zoom [24]. Lastly, participants were provided honoraria as well as financial support for counseling services should they choose to utilize them. Figure 1 provides a schematic of the study design including the creation of focus group cohorts and the schedule of the serial focus groups.

### Analysis

Focus group audio recordings were transcribed verbatim by a third-party transcriptionist and imported into NVivo 12 [25]. We used framework analysis, which involved: **(1)** familiarizing with the transcripts; **(2)** developing a thematic framework by identifying themes emerging from the data, guided by judgments about their meaning, relevance, and significance; **(3)** indexing sections of the data according to specific themes; **(4)** organizing data into charts based on the thematic framework; and **(5)** interpreting the data by mapping concepts, identifying associations, and generating explanations for the findings. We incorporated sex and gender-based analysis (SGBA) to consider the influence of sex, gender, and related factors (e.g., roles, relationships, identities) [26]. Finally, we drew from two relevant frameworks to guide our analysis, particularly with respect to classifying individual-level (e.g., patient) factors and health care system-level enablers that influence disparities in sexual and reproductive health care among AYA cancer patients. At the individual-level, the Campbell and Cochrane PROGRESS-Plus framework outlines social determinants of health (**P**lace of residence, **R**ace/ethnicity/culture/language, **O**ccupation, **G**ender, **R**eligion, **E**ducation, **S**ocial capital, **S**ocioeconomic status, **P**lus age, disability, and sexual orientation, and other personal characteristics associated with discrimination) [27]. At the health care system-level, Andersen’s model of access to medical care outlines predisposing (e.g., education, age, etc.), enabling (e.g., availability of health care facilities), and need (e.g., perceived and/or evaluated need) enablers [28]. Three researchers completed the framework analysis (NO, VC, and PK), and a fourth assisted in resolving conflicts (MADV).

#### Ethics approval and consent to participate

This study received approval from the University of British Columbia’s Behavioural Research Ethics Board (H21-03591) and all participants provided informed consent.

## Results

### Participant characteristics

Our study included 48 AYA cancer patients assigned into eight cohorts who participated in a total of 24 focus groups (Table 1). At the time of study participation, the median age of participants was 33, and at the time of cancer diagnosis, the median age was 31. Participants reported a variety of primary cancer sites, including breast (*n* = 15), colon (*n* = 8), and brain (*n* = 3) and hematological cancers (e.g., leukemia, Hodgkin’s, and non-Hodgkin’s lymphoma) as well as cancers of reproductive organs (e.g., cervical, ovarian, uterine, and testicular). At the time of participation, 68.8% had completed their cancer treatment. In terms of sociodemographic characteristics, most participants identified as White (75%), East and South Asian (6.3% each), and West Asian (4.2%). There was a higher proportion of female (77.1%) and cis-gender women (70.8%), along with representation from non-binary (6.3%) and gender-fluid individuals (2.1%). We had diverse representation of sexual orientation, with the largest groups identifying as heterosexual (64.6%) and bisexual (14.6%), followed by asexual (8.3%), demisexual (4.2%), homosexual (4.2%), and pansexual (4.2%).

**Table 1:** Focus group cohorts and participant characteristics (*N* = 48)^A^

Focus group cohort	Number of participants in each		
Cohort 1: Cis-gender women	10 (20.8)		
Cohort 2: Gender and sexual diverse	7 (14.6)		
Cohort 3: Black, Indigenous, People of Colour	7 (14.6)		
Cohort 4: Cis-gender men	6 (12.5)		
Cohort 5: Diagnosed with breast cancer	6 (12.5)		
Cohort 6: Diagnosed with pelvic cancer	6 (12.5)		
Cohort 7: Diagnosed during adolescence (15–19 years)	4 (8.3)		
Cohort 8: Diagnosed with Stage 4 cancer	3 (6.3)		
**Participant characteristics**			
**Sociodemographic characteristics**		**Cancer characteristics**	
**Age at cancer diagnosis**,** median (range)**	31 (15–39)	**Primary cancer location**,** n**^**B**^	
**Age at participation**,** median (range)**	33 (21–48)	Breast	15
**Race**,** n (%)**		Colon	8
White	36 (75.0)	Brain	4
East Asian	3 (6.3)	Non-Hodgkin’s Lymphoma	3
South Asian	3 (6.3)	Cervical	3
West Asian	2 (4.2)	Thyroid	3
Indigenous x White	1 (2.1)	Ovarian	2
South Asian x West Asian	1 (2.1)	Hodgkin’s Lymphoma	2
South Asian x White	1 (2.1)	Leukemia	2
West Asian x White	1 (2.1)	Sarcoma	2
**Sex assigned at birth**,** n (%)**		Testicular	1
Female	38 (79.2)	Neuroendocrine	1
Male	10 (20.8)	Lung	1
Intersex	0	Spinal	1
**Gender**,** n (%)**		Nasopharyngeal	1
Woman	34 (70.8)	Rectal	1
Man	10 (20.8)	Uterine	1
Nonbinary	3 (6.3)	**Stage**,** n (%)**	
Gender fluid	1 (2.1)	1	8 (16.7)
**Sexual orientation**,** n (%)**		2	11 (22.9)
Heterosexual	31 (64.6)	3	15 (31.3)
Bisexual	7 (14.6)	4	9 (18.8)
Asexual	4 (8.3)	Missing	5 (10.4)
Demisexual	2 (4.2)	**Treatment status**,** n (%)**	
Homosexual	2 (4.2)	Completed	33 (68.8)
Pansexual	2 (4.2)	Currently undergoing	14 (29.2)
**Relationship status**,** n (%)**		Not yet started	1 (2.1)
Married	17 (35.4)		
Single	15 (31.3)		
Common-law or co-habiting	7 (14.6)		
In a relationship	6 (12.5)		
Dating	2 (4.2)		
Separated	1 (2.1)		
**Education**,** n (%)**			
Graduated from a 4-year program	22 (45.8)		
Post-graduate degree	15 (31.3)		
Graduated from a 2-year program	5 (10.4)		
Attended some college and/or university	5 (10.4)		
Secondary or high school	1 (2.1)		
**Religion**,** n (%)**			
No religious affiliation	30 (62.5)		
Christian-protestant	6 (12.5)		
Christian-catholic	4 (8.3)		
Spiritual	4 (8.3)		
Muslim	3 (6.3)		
Jewish	1 (2.1)		
**Income (CAD)**,** n (%)**			
<30,000	5 (10.4)		
30,001–60,000	7 (14.6)		
60,001–90,000	7 (14.6)		
90,001–120,000	15 (31.3)		
120,001–150,000	5 (10.4)		
150,001+	9 (18.8)		
**Location**,** n (%)**			
Ontario	18 (37.5)		
British Columbia	17 (35.4)		
Manitoba	7 (14.6)		
Newfoundland and Labrador	3 (6.3)		
Alberta	2 (4.2)		
Nova Scotia	1 (2.1)		
Student**Employment**,** n (%)**			
Unable to work Employed full-time (40 + hours)	18 (37.5)		
On disability	12 (25.0)		
Student	5 (10.4)		
Unemployed Unable to work	4 (8.3)		
Employed part-time (< 40 h)	3 (6.3)		
Self-employed	3 (6.3)		
Unemployed	3 (6.3)		

^A^One participant was a member in two focus group cohorts

^B^Some participants had more than one primary site of cancer

### Framework Analysis

**Fig. 2:**
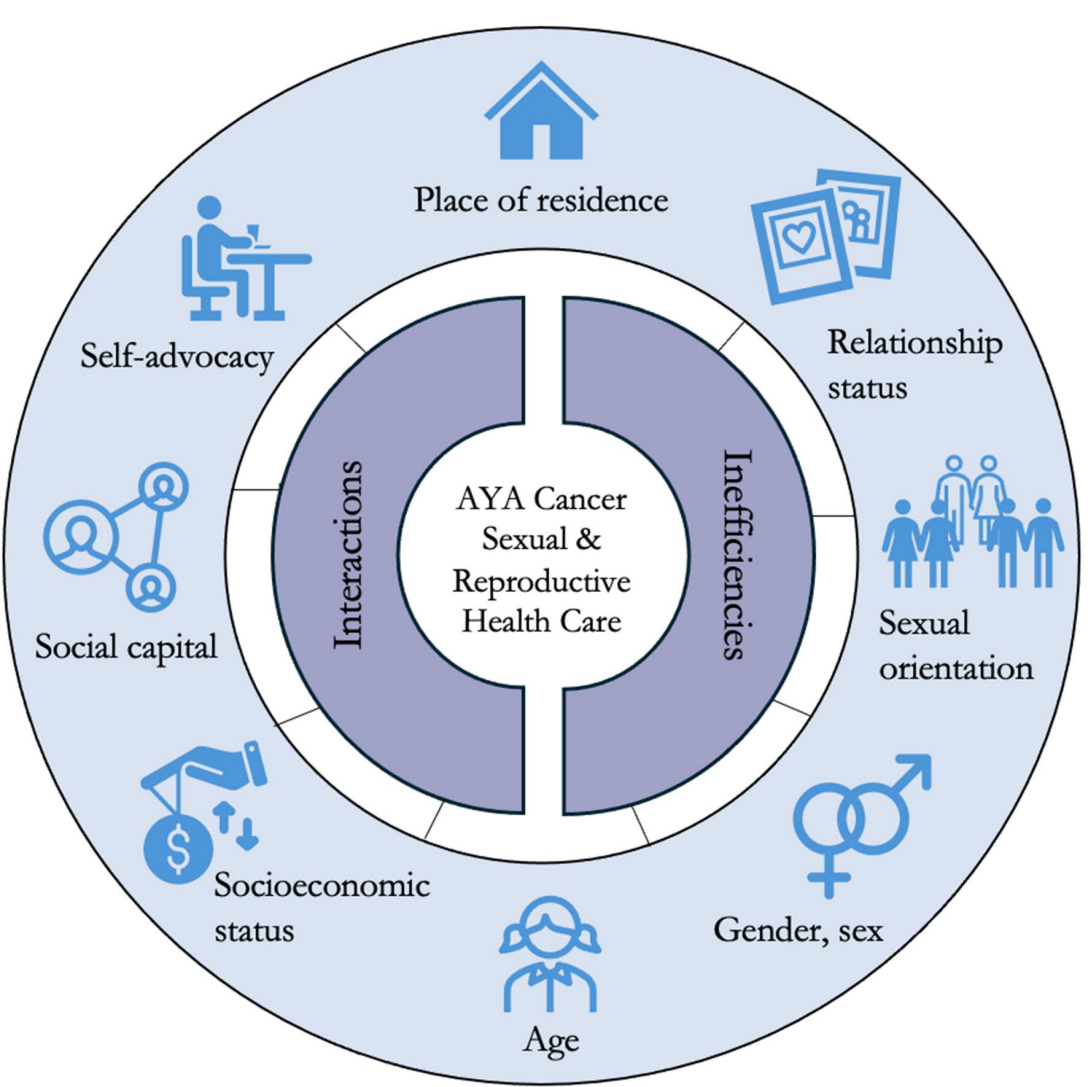
Summary of findings of eight individual-level factors guided by the PROGRESS-Plus (outer circle; **P**lace of residence, **R**ace/ethnicity/culture/language, **O**ccupation, **G**ender, **R**eligion, **E**ducation, **S**ocial capital, **S**ocioeconomic status, and Plus (other personal characteristics associated with discrimination)) framework, and two enablers guided by Andersen’s model of access to medical care (inner circle)

The resultant framework consisted of two themes that captured the experience of AYA cancer patients with sexual and reproductive health care. The **first theme**, on **limited integration of sexual and reproductive health in cancer care** provided insight on the patient experience with the information, supports, and management offered for their sexual and reproductive health care before, during, and after treatment. The **second theme** on **intrinsic and extrinsic influences on sexual and reproductive health care** focused on eight identity (patient-level) factors: place of residence, self-advocacy (occupation, education, language, and more), socioeconomic status, social capital, gender and sex, age, relationship status, and sexual orientation. Additionally, it examined two contextual enablers (health care system-level factors) of inefficiencies and interactions, which influence AYA cancer patients’ experiences with sexual and reproductive health care. Figure 2 provides a visualization of this framework. Each theme is illustrated by participants’ lived experiences as shared in the focus groups.

## Theme 1: limited integration of sexual and reproductive health in cancer care

Participants described varying experiences with access to and provision of information, supports, and management options for both sexual and reproductive health care. **Before cancer treatment**, many experienced anxieties about potential sexual health complications, but noted that “*short term*,* medium term*,* and long term*,* what to possibly expect for both chemo and radiation*,* sexual health did not come up at all.”* This omission was often attributed to the prioritization of life-saving treatment over quality-of-life, with little to no concern for the “*consequences of chemo and all the surgeries… that don’t make [the patient] feel sexually attractive*.” Reproductive health options, such as egg, embryo, and sperm preservation, were more frequently discussed than those for sexual health. However, these discussions were often rushed, with participants feeling that they were not “*given any resources to back up [their] choice”*. They were merely asked *“‘Do you want to do this or not?’ without any discussion of pros and cons or how it would affect [their] treatment”.* One specific example raised during the focus groups was the lack of discussion around potential risks associated with fertility preservation measures, with one participant sharing that they were given only “*30 seconds to make the decision*,* which is irrevocable.”* Another participant reflected on how hurried and impersonal the communication was, explaining, *“It was not really grounded in the context that if it was important for me to have children*,* I probably would’ve been pretty upset with the level of detail that I received.”* One participant eloquently summarized these experiences as being given an “*illusion of choice*”, that is, AYA cancer patients being presented with what appears to be options, but in reality, they do not have the agency in their decision-making.

**During cancer treatment**, lack of attention to sexual health continued to cause stress, particularly for those who were sexually active. As treatment progressed, physical changes such as weight fluctuations, hair loss, and scarring, contributed to a decline in sexual health. Regarding sexual activity, the lack of “*safety guidance*” on the potential exposure risk to partners with chemotherapy made participants feel that an important aspect of their treatment plan was overlooked. There were also instances where treatment plans were changed with minimal notice, introducing risks to sexual health that participants had no time to process, even though patients knew “*it’s not going to be great for [their] sexual health*,* but it would be nice if somebody went over that or talked about some of the different risks that are associated*.” Similarly, concerns about reproductive health persisted, as shown by a participant who underwent a hysterectomy and was left asking, *“‘What’s your plan? Those organs are really important*,* and the hormones they give are really important*,*’ but [they] just got sent home*.” This gap in care also affected participants navigating cancer during pregnancy. One participant, diagnosed with breast cancer while pregnant, shared how they were suddenly told to start chemotherapy because waiting was no longer an option. They noted “*there wasn’t much information out there about how young people going through breast cancer would be affected*,* let alone pregnant young people*” leaving them to make difficult, high-stakes decisions with minimal information and support.

However, there were also instances where the participants themselves, particularly those diagnosed with stage 4 cancers, had deprioritized their sexual and reproductive health during cancer treatment. Sometimes this was intentional, as “*the last thing [they were] thinking of while getting chemo was*,* ‘I’m going to go home and be intimate with my husband.’”* Other times, it was not an intentional choice to deprioritize sexual and reproductive health, but rather the overwhelming nature of the cancer diagnosis and treatment made it difficult for them to focus on anything else. As one participant shared, “*If there were resources offered*,* [they were] deaf to them because it wasn’t on [their] radar…it could’ve been there*,* but it was so far from what [they were] interested in that it didn’t really matter*.”

**After cancer treatment**, the impacts of these gaps in sexual and reproductive health care were even more apparent. One participant described experiencing vaginal atrophy or stenosis and learning too late that “*young people find it helpful to use dilators during chemo*,” but by that point, the damage was irreversible, permanently altering their ability to have penetrative sex. In addition to the physical impacts, participants also shared the emotional impacts from resignation and grief, with getting “*to a point where it just feels like a lost cause and [they] just have to let [sexual health] go”*. Due to their lack of preparation for the lasting impacts of treatment, combined with insufficient follow-up care, participants felt their sexual health was neglected as they *“got information about oral health*,* mental health*,* and digestive health*,* but for some reason*,* sexual health wasn’t a piece of that*.” Regarding reproductive health, female participants reported challenges related to early menopause and received limited guidance on its broader implications, such as osteoporosis, heart disease, and other complications, sharing that “*instead of education or compassion*,* [they] were [just] sent home with a prescription*.” However, many others also reported receiving limited guidance on the use of hormone replacement therapy.

## Theme 2: intrinsic and extrinsic influences on sexual and reproductive health care

### Identity (patient-level) factors

#### Place of residence

Patients living in urban areas, where larger hospitals and cancer centers were located, generally had access to more resources, while those in rural or smaller towns faced very limited options. In smaller towns or rural areas, the specialized “*resources such as sex therapy or pelvic therapy just didn’t exist within the region*,*”* and “*parents had to drive [them] to the clinic…other people in other cities who had to find accommodations nearby [were in] much harder circumstances.”* Socioeconomic status often intersected with geography, as those with the means to travel and arrange for accommodations at urban centers were able to access sexual and reproductive health care.

#### Self-advocacy (Occupation, education, Language, and Plus)

Participants found self-advocacy as a method to access appropriate sexual and reproductive health care, especially those who were educated or had recently navigated the medical system. Education played a pivotal role in patients’ ability to navigate the complexities of cancer care and self-advocate. Participants who were comfortable with medical journals “*started doing [their] own research”* regarding fertility treatments *“and wondered ‘why would [they] waste all this stress and time and money freezing embryos if it’s a 60% chance they’ll survive post-thaw’”*? One participant noted, their *“master’s degree and being White*,*”* gave them the ability to advocate for themselves and, “*and do [their] own research”* that led to better outcomes than someone with “*English as a second language”* or less familiarity with the health care system. Participants shared that *“if [they] didn’t ask the questions and weren’t super particular about it*,* [they] don’t think [their fertility care] would’ve happened.”* Additionally, recent health conditions or experiences within the health care system positioned patients to better advocate for themselves. A colorectal cancer participant with a “*recent family experience with ovarian cancer proactively asked for a fallopian tube removal”* during an unrelated surgery, and appreciated how their health care providers “*took [their advocacy] seriously and didn’t brush them off.”* While these stories underscore how knowledge, confidence, and previous experiences in the health care system allowed some AYA cancer patients to better advocate for their sexual and reproductive health, they also highlighted the disparity faced by those without such backgrounds, leaving them more vulnerable to inadequate care and overlooked needs.

#### Socioeconomic status

Socioeconomic status significantly impacted access to sexual and reproductive health care for AYA cancer patients and often created barriers for those unable to afford this care. Reproductive health was a major area of cost for AYA cancer patients. Although participants “*thought if [they] were to ever get something like cancer*,* [fertility preservation and family planning] would be covered*,*”* in actuality, the “*finances and cost of reproductive health care are extreme…it’s a real barrier.”* Although some participants lived in provinces that covered sperm and/or egg retrieval, many still faced associated costs for subsequent procedures, as *“insurance covers the drugs*,* but it doesn’t cover the surgeries or procedures*,* so you’re looking at $15*,*000*,* $20*,*000*,* $25*,*000 with no guarantee of success.”* Care to support sexual health was also costly. Some participants who had access to private insurance shared this as an importance resource to pay for care such as pelvic floor therapy. Unfortunately, many AYA cancer patients did not have private insurance, making them reliant on inconsistent and often inadequate public funding programs. Further complicating access to these programs, many funding options described were based on reimbursement, requiring patients to *“have a credit card with a high enough limit to pay for it*,*”* which was a significant hurdle for many AYA patients who were often less financially established than older cancer patients.

#### Social capital (Plus)

The presence of social network during appointments represented social capital that can serve as both a facilitator and a barrier to receiving optimal sexual and reproductive health care for AYA cancer patients. For some participants, family members took notes, collected handouts, and assisted in processing complex medical information. In some cases, family members were also advocates as shared by one participant whose family member, a lawyer, intervened when a provider missed a significant diagnosis. The quote *“you shouldn’t have to have a freaking lawyer in your family to get the care you need”* underscored how socioeconomic status and family members’ professional backgrounds influenced care, and highlighted inequity for those without access to similar privileges.

However, the presence of family during appointments also introduced complications. A participant shared that their parents asked questions that “*wasted precious [appointment] time”* especially if these were information that the participant already knew. Another participant described the discomfort of discussing *“if [they] could engage in certain sexual activities with their [parents] right next to them”*. Collectively, the possible discomfort or reluctance that emerged when discussing sexual and reproductive health in the presence of family impeded conversations during appointments that were already short.

#### Gender and sex

Gender and sex played a significant role in shaping the ease or difficulty of accessing both sexual and reproductive health care, especially around sensitive topics like fertility preservation and sexual well-being. For participants assigned male at birth, the process of sperm banking was described straightforward and non-invasive, but for those assigned female at birth, fertility preservation through egg retrieval was much more physically demanding and invasive. One participant noted how providers “*didn’t really care about [their] pain*” during these procedures, reflecting a broader trend in dismissing women’s discomfort, and that “*they’re just like*,* this is what [the] vaginas for. Things are supposed to go in it.”* The lack of proactive discussions around sexual health for women was a recurring issue; the physical effects of treatment, such as vaginal dryness due to surgical menopause, were often ignored unless explicitly asked about, leaving many without crucial support. Many women participants highlighted how having men as health care providers “*definitely impacted the awkwardness of bringing up [sexual health]”*, and that they never initiated conversations about fertility preservation, leaving the burden on the patient to “*ask if the chemo was going to affect [their] ability to have children.”* In contrast, some women participants did recount how their all-women care team took their concerns about fertility preservation seriously, explaining, *“my surgeon*,* the residents*,* the anesthesiologist*,* and the nurses were all women… it made me feel so comfortable.”*

For patients receiving gender-affirming care, such as trans and nonbinary individuals, the challenges were even more significant. A trans-man participant shared how their cancer diagnosis was delayed because a physician dismissed their breakthrough bleeding, which was “*one of the major signs that [they] had cancer.”* As symptoms related to gender-affirming treatments may be conflated with symptoms of cancer, this leaves trans patients vulnerable to delays in both diagnosis and treatment. As current health care guidelines for trans folks are noncomprehensive, participants highlighted the broader issue, noting that *“when you’re screening for any type of cancer with trans people*,* it ends up being a bit of a guessing game.”* However, when health care providers were knowledgeable and inclusive, the care experience was different. One participant expressed gratitude for their treatment team, particularly their family doctor, who *“saw trans patients and had a huge amount of knowledge… [the doctor] was able to talk to [them] about fertility preservation in the same breath as testosterone use.”* This highlighted the positive impact of having health care providers who were both well-versed in trans health and willing to have sensitive discussions about sexual and reproductive health.

#### Age (Plus)

Age was a significant barrier for AYA cancer patients, as health care systems tend to cater either to older populations or to pediatric patients, leaving those in between underserved. Research on cancer treatments is an area where age becomes a barrier for AYA patients, as *“all of the studies that have been published are for women above the age of 60*,*”* underscoring the absence of research tailored to younger patients, especially those facing premature menopause or sexual quality of life concerns. “*When [participants] ask [their] gynecologist for additional reading”*, they’re told “*we have research*,* but it doesn’t pertain to you… colon cancer isn’t really a cancer of young people.’”*, even though they *“have different needs that are never reflected in the medical care system”* This age gap left younger adult patients feeling “*like a trendsetter”*, especially in smaller communities where expertise for AYA cancer patients was so limited that a participant noted their “*oncologist only had one other woman [their] age try for a baby*,* so she didn’t have the statistics*.” Indeed, resources for older cancer patients, while plentiful, often fail to address the concerns of younger adults. One participant explained how they are “*the youngest one at support groups”*, where *“everyone is talking about being lucky to have grandkids*,* and nobody can relate when [they] share [their] story.”* The timing of support groups, often held during the day when younger adults are working or with family, further excluded those who “*can’t retire yet*,* so can’t join them*”.

#### Relationship status (Plus)

Not being in a relationship lead to significant gaps in both sexual and reproductive health care for AYA cancer patients, as providers often assumed there were no concerns if a patient is single. Participants noted that *“when they asked [them] if they have a sexual partner and [they] said no*,* [they] never received any sexual health support again.”* However, *“do you have to have a partner for that to be something that’s important? You can be a sexual person without a partner as it turns out*,*”* highlighting that sexual health concerns (such as reduced drive or function) should still be prioritized, regardless of relationship status. This lack of sexual health guidance left some participants in awkward situations, such as having to call their oncologist’s secretary to ask, *“Is it okay if I masturbate now?”* after surgery, stressing a broader lack of proactive discussion around AYA cancer sexual health needs.

These presumptions not only ignored sexual health concerns but also resulted in reproductive health issues being unaddressed. For instance, single patients felt even more alienated from reproductive health research and support programs that prioritized couples. One participant noted, *“a lot of the research studies were for people and their partners*,* and it made me as a person who was single not feel comfortable.”* This emphasis on couples may keep single patients out of important conversations about options for reproductive health or fertility preservation. Participants also found it *“so hard to date when [they’re] going through treatment and even after… and there’s literally no support for any of that at all*,*”* highlighting the unique sexual and reproductive health challenges faced by single AYA patients, who often struggle with these conversations when dating.

#### Sexual orientation (Plus)

Sexual orientation presented a unique set of challenges in accessing sexual and reproductive health care, particularly for queer AYA cancer patients who encountered heteronormative assumptions from health care providers. For reproductive health, many participants expressed frustration at how the health care system frequently centered heterosexual relationships during fertility treatments, often pushing for embryo freezing as opposed to eggs or sperm, and being “*constantly asked where [their] husband was during fertility treatments*,*”* leaving queer patients feeling unseen and “*really fed up with the heteronormative assumptions”*. Indeed, participants stated that assumptions about their sexuality led to moments of inappropriate sexual health cancer care. One participant shared their frustration with constantly being asked “*if [they’re] pregnant every time [despite] being gay*,* and [they weren’t] having sex*,* but they never believed [them]”.* These interactions not only delayed timelines for treatment but also made queer patients feel their sexual orientation was disregarded in their reproductive health care. These assumptions continued for sexual health care, as it “*was very much about penetrative sex. It was like*,* make sure you wear a condom… but no one talked about other forms of intimacy”.* This limited focus on heterosexual norms left out vital conversations about safer sex and intimacy for those in same-sex relationships or for people whose sexual practices did not align with heteronormative assumptions. In contrast, a participant reflected on the comfort they found in a therapist who shared their identity as a queer person, and that having *“a therapist who is also queer has opened a lot more in our conversations… [they] feel a lot safer to explore those topics with her.”*

### Contextual enablers (health care system-level factors)

#### Inefficiencies (that limited access to sexual and reproductive health care)

Participants shared that their limited access to sexual and reproductive health care largely resulted from delayed discussions, rushed appointments, unclear roles among providers, and long wait times. Many patients felt that discussions about sexual and reproductive health “*were brought up too late*,*”* often because *“young adult cancers are unfortunately diagnosed much later*,*”* so there is little time before treatment begins to consider fertility preservation or sexual quality of life. Appointments were usually only 15 min long and often rushed, as *“doctors would usually come in and get out as fast as possible*,* just explaining what’s going on and what will happen*,*”* leaving patients without *“a chance to sit and ask questions about [their sexual and reproductive health].”* Participants also felt that health care providers were unsure whose responsibility it was to manage a patient’s sexual and reproductive health. For those who managed to obtain referrals to sexual and reproductive health specialists, long wait times were common, often resulting in delays in seeing a provider after cancer treatment. This left them questioning, *“what’s the point? I’m going to be waiting about a year to even ask these questions*,* and by then*,* my situation may have changed*.”

#### Interactions (that shaped experience with sexual and reproductive health care)

Interactions with the health care system profoundly shaped AYA cancer patients’ experiences with their sexual and reproductive health care. Participants often described these interactions as being affected by insensitivity, stigma, and medicalization of their bodies.

Participants felt that health care providers often lacked sensitivity in their interactions, examples included telling a participant at age 16 that they “*might not be able to have kids*” without the chance to absorb or ask questions, telling a couple to “*use it or lose it*” or “*just put some lube on it”* when asking if it is safe to have sex again, and asking *“Is it a bad thing?”* when a patient observed that their periods had stopped at age 32. Fertility preservation with fertility clinics, for females in particular, was often described as “*sales-y and pushy*,” as if “*they were pressuring [them] to do it for money*”. With little attention paid to the fact that for cancer patients, fertility preservation was often a last resort, not an immediate goal. The insensitivity of these interactions was further exacerbated by the handling of critical information, such as the viability of embryos. “*The number of embryos started big and slowly got less and less. The nurse just called and said*,* ‘Yep*,* this is how many there are*,* bye.’ I was devastated*.” Several participants noted the insensitive physical spaces, such as clinics “*plastered in pictures of adorable babies”*, which only heightened the emotional toll for those who either had no interest in having children or were grappling with infertility.

Quite notably, stigma was a major influence on the interactions surrounding sexual health care. Participants found that health care providers were ill-equipped to handle conversations regarding sexual health, that *“[the health care provider] couldn’t talk to it at all…she actually seemed really embarrassed.”* One participant reflected on how discomfort in discussing sexual health might stem from how “*no matter how old we are*,* we as society can be squeamish around talking about sex*,* even in a very clinical way.”* They suggested that this discomfort leads to rushed or avoided conversations about sexual health, such as, *“Are you having sex? Are you still able to? Is there anything you need to talk about? Oh*,* okay*,* we’re gonna move on right now.”*

Beyond conversations, patients felt that certain assessments and procedures made their “*body feel medicalized”*, which also exacerbated previous sexual traumas. Participants described undergoing invasive exams such vagina and rectal exams with medical students observing, without being informed of their presence beforehand. This left participants *“feeling uncomfortable”* and so unsafe that they *“just wanted to hide underneath [the] sheets in the dark*,* but [they couldn’t].”* For those with a history of sexual trauma, these interactions “*brought back the feeling of loss of control of [their] body*”. Thus, a more patient-centered approach is necessary for the planning and delivery of these exams.

## Discussion

Using serial focus groups that fostered trust and community building, our study provides key insights into the sexual and reproductive health care experiences of AYA cancer patients. Participants described their difficult experiences with sexual and reproductive health information, support, and care provided before, during, and after cancer treatment. Notably, we identified a range of factors – eight at the individual level and two related to health care systems – that shape these experiences. The findings underscore the importance of validating AYA cancer patient experiences with highly sensitive and often stigmatized issues such as sexual and reproductive health, particularly in the face of a life-altering diagnosis like cancer. Moreover, these insights have the potential to guide patient-centered, intersectional strategies to fill in sexual and reproductive health care gaps by addressing individual and health care facilitators and barriers faced by AYA cancer patients.

We used the PROGRESS-Plus framework to consider how individual-level factors impact AYA cancer patient experiences with sexual and reproductive health care. This approach is necessary, as outcome disparities based on individual-level factors are prevalent in this patient population [14, 15]. We note that our findings related to age, socioeconomic status, self-advocacy, and geographic location add to a growing literature on what prevents full realization of excellent AYA cancer care [29]. Further, our study found differences in sexual and reproductive health care experiences based on sex, gender, and sexuality, with an overt focus on oncofertility and reduced integration of sexual quality of life. These findings impact the patient experience - Bentsen et al. (2021) interviewed 12 female AYA cancer patients about their reproductive health care experiences during treatment. Females highlighted difficulties in accessing reliable fertility information, a lack of adequate support, and feelings of mistrust stemming from confusing or insufficient communication [12]. Similarly, 2023 study by Ussher et al. completed online surveys with 95 (31 non-cis and 91 non-heterosexual) and interviews with 19 (9 non-cis and 19 non-heterosexual) AYA cancer patients and found that participants felt that cancer was “disrupting their developing identities and involvement with LGBTQI communities”, they faced “internalized prejudices that impacted identities”, they felt “invisible in cancer care” due to “cis-heteronormative assumptions,” and experienced “precarious social support” in cancer networks. Ussher’s findings reflect ours in that AYA cancer intersectionality with sex, gender, and sexuality result in impacted outcomes and care, undoubtedly for sexual and reproductive health as well.

An important finding in our study was participants’ insights of limited or lack of support for their sexual and reproductive health care due to limitations with the health care system. There are limited studies that have found similar findings [13, 30]. A study by Albers et al. in 2022 conducted interviews with six doctors and eight nurses in AYA sexual health care and discovered that providers are unclear whose responsibility it is to bring up sexual health, required optimal timing to discuss this with the patients, needed more education to enable these conversations, and necessitated more informative material for AYAs [30]. Similarly, in 2019, Frederick et al. conducted semi-structured interviews with 23 AYA cancer patients (ages 15–25), using thematic analysis to examine their experiences with sexual and reproductive health communication in oncology care [11]. This study found that AYA cancer patients face significant gaps in sexual and reproductive health communication with oncology clinicians. Interviews revealed barriers like patient discomfort and lack of clear guidance, highlighting the need for better clinician training and education to improve sexual and reproductive health discussions. Overall, it is clear that health care systems have not provided providers with adequate time, resources, or education to fully realize the necessary sexual and reproductive health care needed for AYA cancer patients. To close this gap, we and others suggest increased education for providers, reducing wait-times by integrating dedicated AYA providers, and providing relevant resources and programming for patients [13, 29]. These suggestions should be designed with a patient-oriented, intersectional lens that takes into account the individual-level facilitators and barriers identified in this study such as age, sex, gender, and sexual orientation [31]. 

A key strength of our study is the original use of serial focus groups, which fostered a sense of safety and community among participants while encouraging peer-to-peer knowledge sharing, as reflected in the high retention rate throughout the sessions. Furthermore, using an online approach for recruitment and data collection increased accessibility. Also, targeted recruitment allowed us to capture a diverse range of participants across both demographic and clinical characteristics. Our patient-oriented approach through inclusion of patient research partners – from study design (e.g., developing and testing the topic guide), to data collection (e.g., co-facilitating focus groups), to data analysis – added valuable insights. However, a potential limitation is that the sensitive nature of the topics may have made some participants hesitant to discuss certain issues openly with others in the group.

Altogether, our study found that sexual and reproductive health care is impacted for AYA cancer patients, especially through system- and individual-level SDH factors. We suggest that a one-size-fits-all approach is ineffective for improving outcomes and experiences. Instead, an intersectional perspective is required.

## Electronic supplementary material

Below is the link to the electronic supplementary material.

Supplementary Material 1

## Data Availability

Availability of data and material: The data underlying this article will be shared on reasonable request to the corresponding author.
