# Supplementary material for: What factors influence sexual and reproductive health care among adolescent and young adult cancer patients?: a novel serial focus group study
Source: BMC Cancer. 2025 Jul 1;25:1134. doi: 10.1186/s12885-025-14380-w (PMC12218939; doi:10.1186/s12885-025-14380-w)
Supplement: Supplementary file 1 — Supplementary Material 1 [file 12885_2025_14380_MOESM1_ESM.docx]

**Topic guide for focus groups**

**Focus group #1 questions (Intro and reproductive health):***

*General*

- What does sexual and reproductive health mean to you?

*Before treatment*

- What information did you receive about potential impacts on your reproductive health before undergoing cancer treatment?

*During/After treatment*

- How has your cancer and its treatment affected any aspect of your sexual and reproductive health?
  - Look for outcomes (fertility/family planning)
  - Look for changes across varying stages and treatment levels
  - *Were there any impacts on your romantic relationships?*
- How have changes in your reproductive health with cancer impacted your self-esteem? Your sense of self?
  - *What was the mental health impact of these changes?*
  - *How has this changed over time?*
- How has your perspectives and expectations of reproductive health changed over time?
  - *Would you feel differently about that now?*
- How have your reproductive and family planning goals changed over time?
  - *Were there changes in your timelines and priorities?*
- Are there any stories you feel comfortable sharing that captures your experiences with your reproductive health?

**Focus group #2 questions (sexual health):***

*Before treatment*

- What information did you receive about potential impacts on your sexual health before undergoing cancer treatment?

*During/After treatment*

- How has your cancer and its treatment affected any aspect of your sexual health?
  - Look for outcomes (sexual function/sexual quality of life)
  - Look for changes across varying stages and treatment levels
  - *Were there any impacts on your sexual and/or romantic relationships?*
- How have changes in your sexual health with cancer impacted your self-esteem? Your sense of self?
  - *What was the mental health impact of these changes?*
  - *How has this changed over time?*
- How has your perspectives and expectations of sexual health changed over time?
  - *Would you feel differently about that now?*
- Are there any stories you feel comfortable sharing that captures your experiences with your sexual health?

**Focus group #3 questions (health care system and the future):***

*Could-haves*

- What were your interactions with the health care system (e.g. health care providers, nurses, etc.) about sexual and reproductive health and cancer? How did that go?
  - *Did you use alternate providers (physiotherapist, occupational therapist, naturopathy), and how did this impact your outcomes?*
  - *Were your experiences and beliefs considered?*
- Were there any career or financial impacts to the changes in your sexual and reproductive health?
  - Contextualize this (opportunity cost)
  - *Additional financial burden/need for support?*
- Are there factors that you felt impacted your access to appropriate and available sexual and reproductive health care?
  - E.g., Sex, gender, race, rural location
- What role did/does a support system play for your sexual and reproductive health and cancer?
  - *What does a supportive community look like for you?*
- If you had been fully informed of the sexual and reproductive health risks, what would be different for you now?
- What do you think is the role of the health care system in supporting your sexual and reproductive health before, during, and after cancer treatment?
  - *What would need to happen to facilitate this change?*
  - Emphasize gaps
- What would be the best way/time for you to receive information or support about your sexual and reproductive health?
- When you think of your journey with your sexual and reproductive health, what you would tell someone who is going to start cancer treatment?
- What prompted you to participate in this research?

** For all questions, they will only be asked if the information does not come up in the conversations initiated from previous questions*
